# Supplementary material for: Polyester degradation by soil bacteria: identification of conserved BHETase enzymes in Streptomyces
Source: Commun Biol. 2024 Jun 12;7:725. doi: 10.1038/s42003-024-06414-z (PMC11169514; doi:10.1038/s42003-024-06414-z)
Supplement: Supplementary file 2 — Supplementary Information [file 42003_2024_6414_MOESM2_ESM.pdf]

Supplementary Table 1

Table 1: Halo formation of Streptomyces strains in bulk screening on BHET and different agar brands. the + symbol represents halo formation. – symbol represents growth but no formed halo and ng stands for no growth.

Agar	StrepMM BHET	StrepMM +Glcnac	Difco	StrepMM BHET	StrepMM +Glcnac	IB	StrepMM +Glcnac
MBT1	+	+		+	+		+
MBT2	+	+		+	+		+
MBT3	+	+		+	+		+
MBT4	-	-		-	-		-
MBT5	-	+		+	+		+
MBT6	-	-		+	+		+
MBT7	-	-		-	-		-
MBT8	-	-		-	-		+
MBT9	-	-		-	-		+
MBT10	ng	-		-	-		-
MBT11	+	-		-	-		+
MBT12	+	+		+	+		+
MBT13	-	-		-	-		-
MBT14	-	-		-	-		ng
MBT15	-	+		+	+		+
MBT16	-	-		-	-		-
MBT17	-	-		-	-		-
MBT18	+	+		+	+		+
MBT19	-	-		-	-		-
MBT20	-	-		-	-		ng
MBT21	-	-		-	-		+
MBT22	-	-		-	-		+
MBT23	-	-		-	-		+
MBT24	-	-		-	-		+
MBT25	-	-		-	-		-
MBT26	-	-		-	-		-
MBT27	-	-		-	-		-
MBT28	+	+		+	+		+
MBT29	+	+		+	+		+
MBT30	-	-		-	-		-

MBT31	-	-	-	~	~
MBT32	-	-	-	-	-
MBT33	+	~	+	~	+
MBT34	-	-	-	-	+
MBT35	-	-	-	-	-
MBT36	ng	ng	ng	ng	ng
MBT37	-	-	-	-	-
MBT38	+	+	+	+	+
MBT39	+	+	+	+	+
MBT40	-	-	-	-	-
MBT41	-	-	-	-	~
MBT42	-	-	-	-	-
MBT43	-	-	-	-	~
MBT44	-	-	-	-	-
MBT45	+	-	+	-	-
MBT46	+	-	+	-	-
MBT47	+	+	+	+	+
MBT48	-	-	-	-	-
MBT49	-	-	-	-	-
MBT50	~	+	-	+	+
MBT51	~	-	-	-	-
MBT52	-	-	-	-	-
MBT53	-	-	-	-	-
MBT54	-	-	-	-	-
MBT55	-	-	-	-	-
MBT56	-	-	-	-	-
MBT57	-	-	-	-	-
MBT58	-	-	-	-	-
MBT59	-	-	-	-	-
MBT60	-	-	-	-	-
MBT61	-	-	-	-	-
MBT62	+	+	+	+	+
MBT63	-	-	~	-	-
MBT64	~	-	+	-	-
MBT65	+	+	+	+	-
MBT66	+	-	+	-	-
MBT67	-	-	-	-	-

MBT68	ng	-		-	-		-
MBT69	-	-		-	-		ng
MBT70	+	+		+	+		+
MBT71	-	-		-	+		-
MBT72	-	-		-	-		-
MBT73	-	-		-	-		-
MBT74	-	~		-	-		-
MBT75	-	-		-	~		-
MBT76	ng	ng		ng	ng		ng
MBT77	-	-		~	-		-
MBT78	-	-		-	-		-
MBT79	+	+		-	~		~
MBT80	+	-		~	-		-
MBT81	-	+		~	-		-
MBT82	-	-		-	~		-
MBT83	ng	ng		ng	ng		ng
MBT84	ng	ng		ng	ng		ng
MBT85	+	+		~	+		~
MBT86	~	~		~	~		-
MBT87	-	-		-	-		-
[redacted]							
MBT89	+	+		+	+		+
MBT90	-	-		-	-		-
MBT91	+	+		+	+		+
MBT92	+	+		+	+		+
MBT93	+	+		+	+		~
[redacted]							
MBT95	ng	ng		ng	ng		ng
MBT96	-	-		-	-		-

Supplementary Figure 1

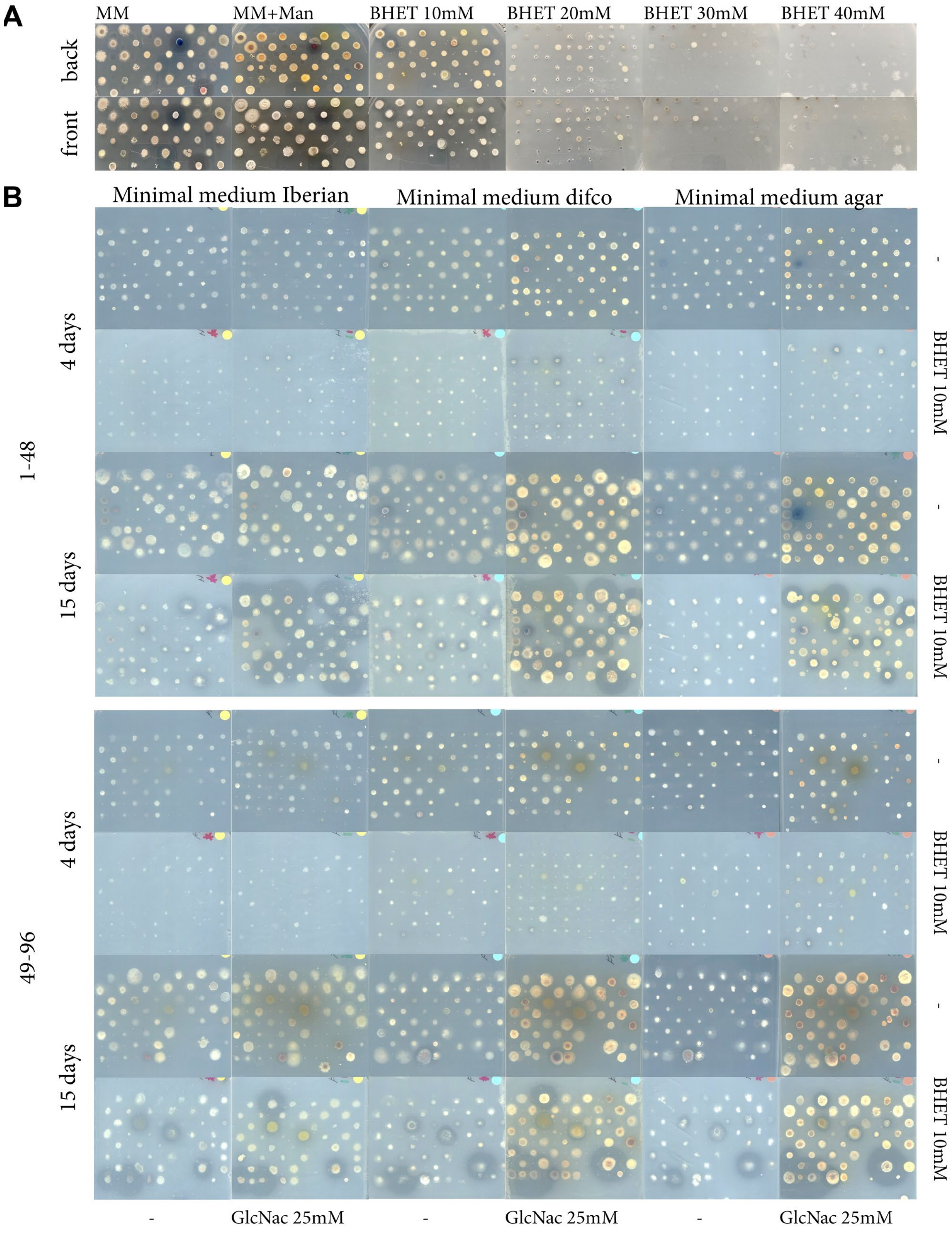

Figure 1: ***Actinobacteria screens on BHET.***
A) Toxicity screen of 36 Actinobacteria on BHET concentrations ranging from 0 to 40mM of BHET. B) Bulk

screens of 96 Actinobacteria on StrepMM Iberian agar, Difco agar and agar agar with and without N-acetyl glucosamine as inducer at 4 and 15 days of growth.

A bulk screen for BHET degradation was performed with all 96 strains split over two 96-well plates to provide them with enough space for development. Seven strains did not grow on the plates and were taken out of the screen. Spore suspensions were stamped onto plates containing StrepMM with different agar brands containing combinations of BHET [10 mM], Mannitol [25 mM] and GlcNAc [25 mM]. The type of agar used clearly impacted the observed degradation patterns. The agar brand seems to have a clear influence on the degradation pattern (Fig. S1B). Difco agar was chosen as the agar source for all further experiments since most strains showed growth on Difco agar. The addition of GlcNAc resulted in a different halo pattern and more predominant halos during the bulk screen and induced BHET degradation in most strains (Fig S1, Supplement 1).

Supplementary Table 2

Table 2: Schematical representation of the halo formation of the MBT strains on BHET in different conditions.

strain	StrepMM BHET	BHET+man	BHET+GlcNAc	BHET GlcNAc+man
1	-	+	+	+
2	-	+	+	+
3	+	+	+	+
5	+	+	+	+
6	-	-	-	-
8	-	-	-	-
11	-	-	-	-
12	+	+	+	+
13	-	-	-	-
15	+	+	+	+
18	+	+	+	+
21	-	-	-	-
25	-	-	-	-
28	-	+	+	+
29	-	+	+	+
31	-	-	-	-
33	-	-	-	-
37	-	-	-	-
38	-	+	+	+
39	-	-	+	+
45	-	-	-	-
47	-	+	+	+
50	-	-	+	+
62	-	-	-	-
65	-	-	-	-
70	-	-	-	-
71	-	-	-	-
76	-	-	-	-
79	-	-	-	-
80	-	-	-	-
81	-	-	-	-
85	-	-	-	-
86	-	-	-	-
89	+	+	+	+
91	-	+	+	+
92	+	+	+	+
93	-	-	-	-
96	-	-	-	-
S.coelicolor	-	-	-	-

Supplementary Figure 2

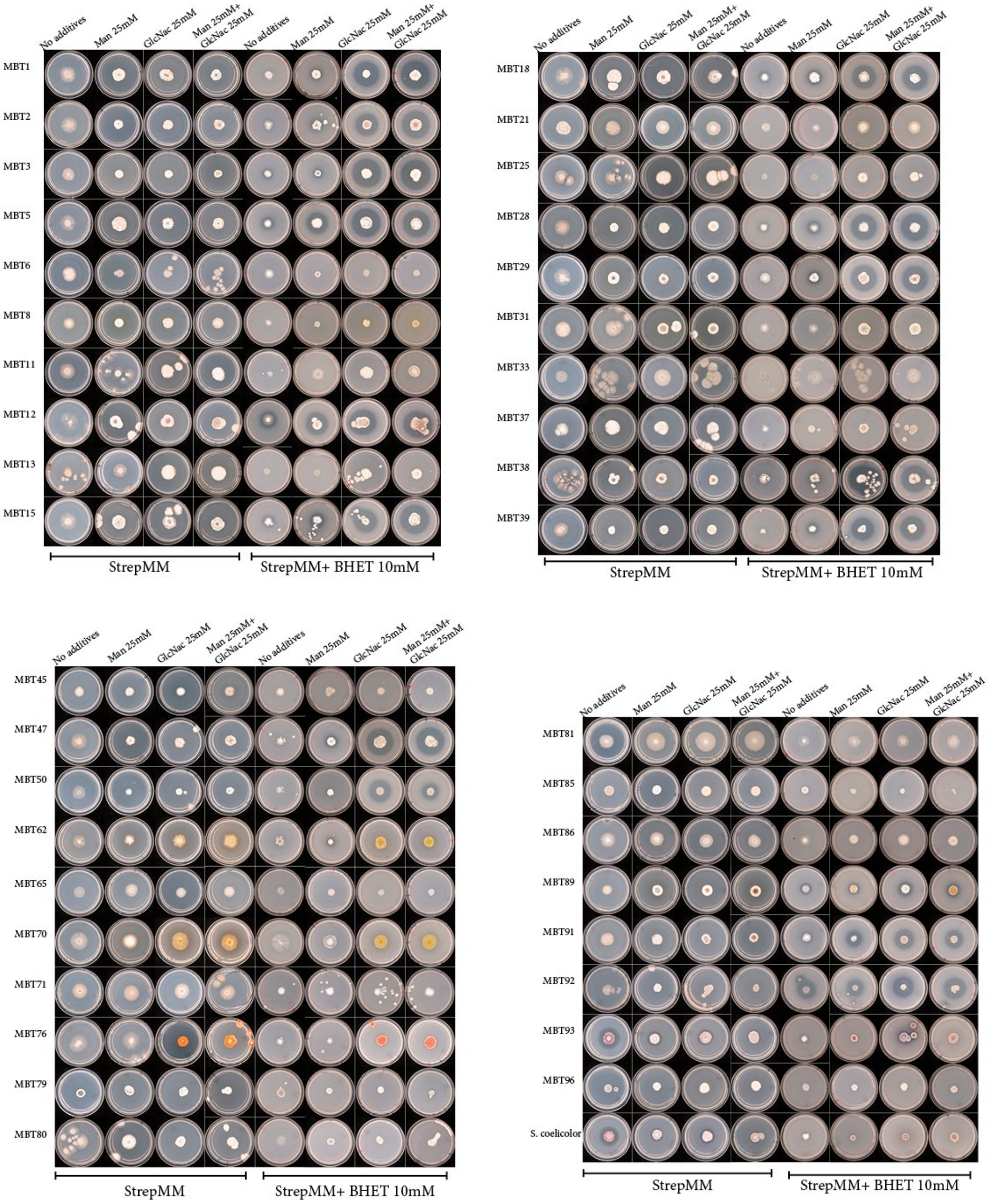

*Figure 2: **Individual screening of Streptomyces strains***

Individual screen ofl active strains on Strep MM difco agar with and without Mannitol, BHET and GlcNac after 10 days of growth.

Supplementary Figure 3

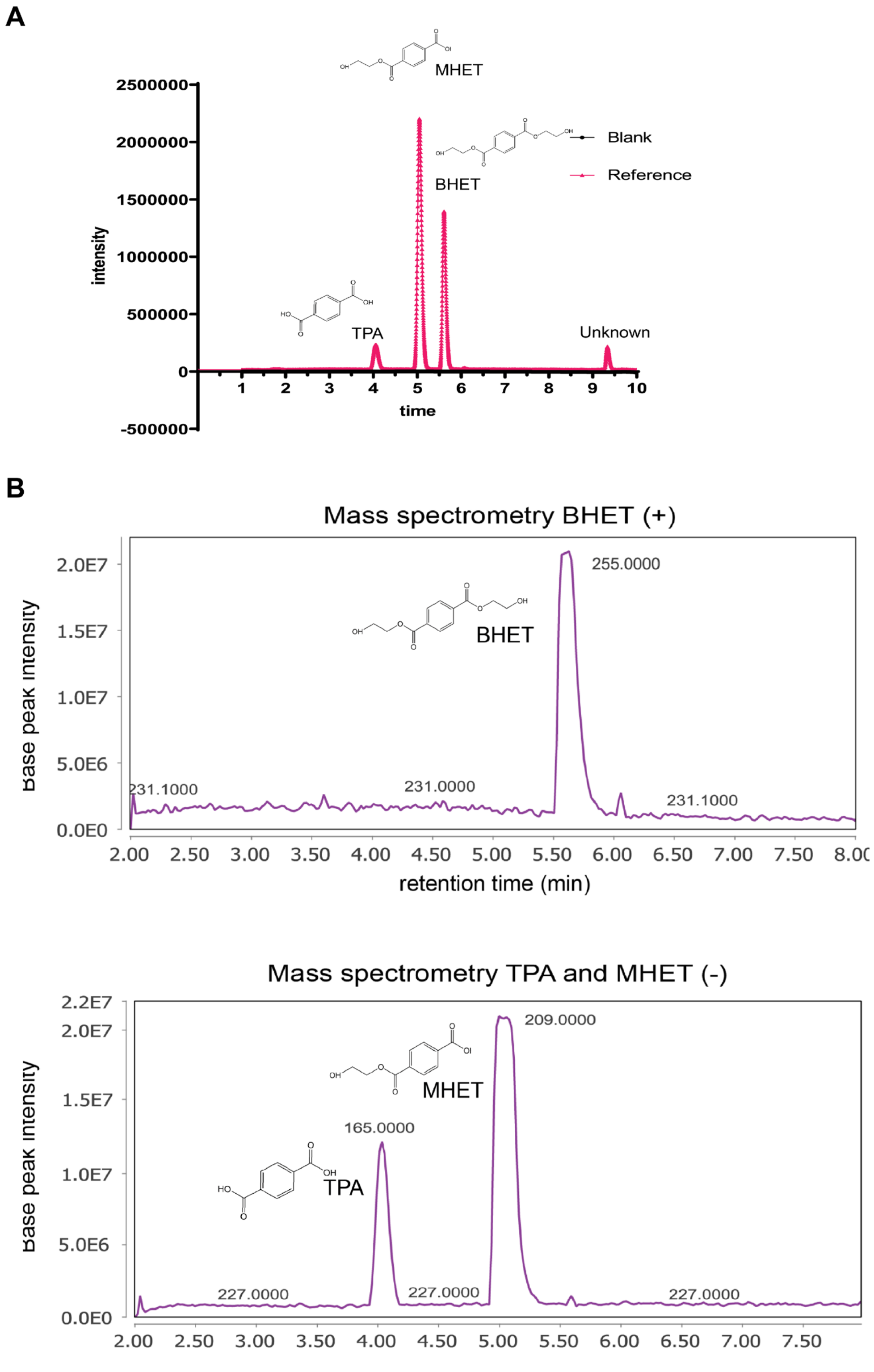

Figure 3: ***LC-MS analysis of BHET, MHET and TPA***
B) LC-MS trace of BHET in positive ionization, MHET and TPA in the negative ionization. C) The UV spectrum at 240 nm of TPA, MHET and BHET with corresponding retention times.

In the negative ionization mode, the mass of TPA (166 g/mol) could be observed around 4 min retention time showing a small peak on the UV spectrum, MHET (210 g/mol) appeared around 5.2 min with a strong signal on both the MS as well as the UV. Finally, BHET (255 g/mol) could be observed in the positive ionization mode between 5.5 and 6 min with a strong signal at 240 nm (Fig. 2B and 2C). Around 9.5 min, impurity was observed, which was present in the BHET stock (Fig. 2C).

Supplementary Figure 4

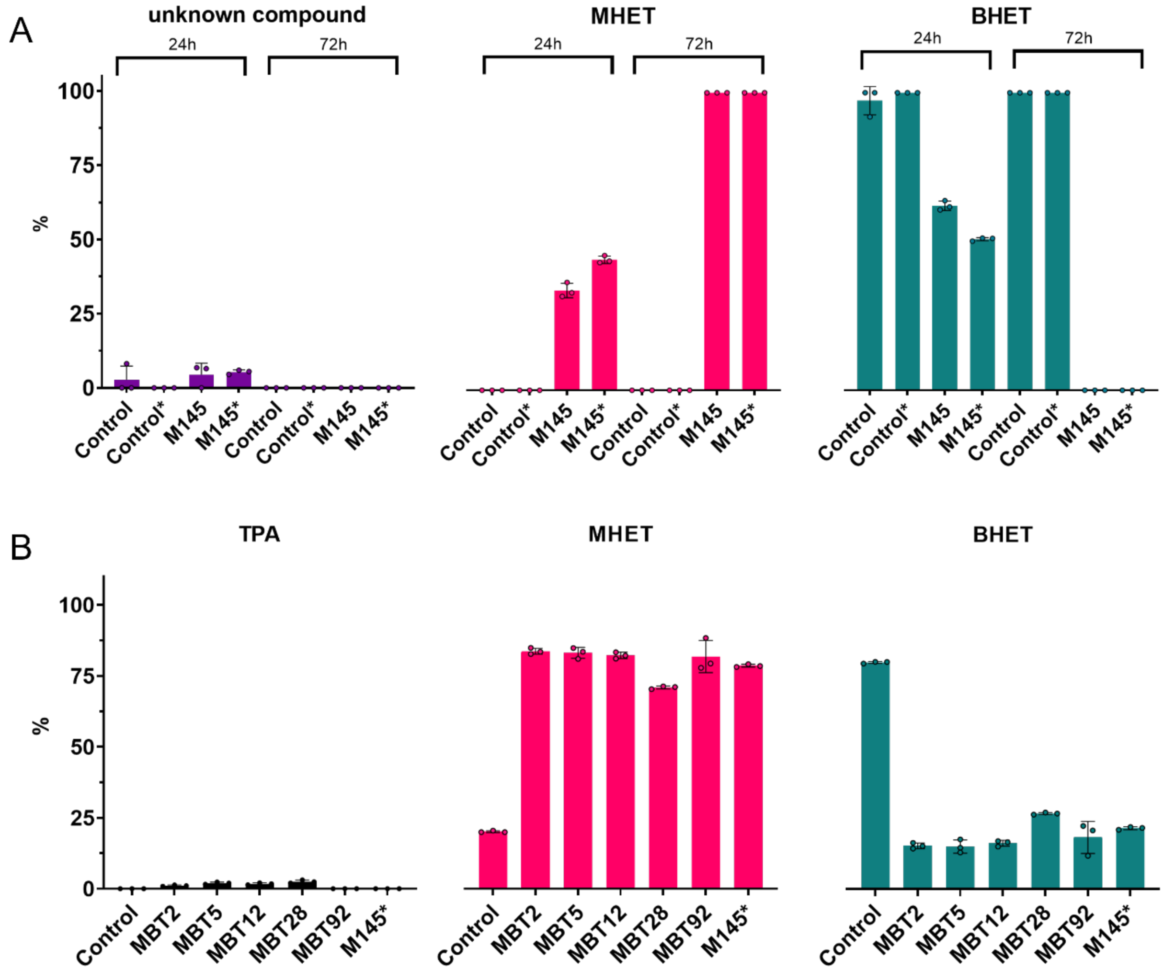

Figure 4: ***The ability of S. coelicolor M145 and other Streptomycetes to degrade BHET.***
*A) The degradation of BHET by S. coelicolor in liquid medium (n=3). The control samples are displayed as control and only contain NMM with BHET [10 mM]. The addition of GlcNAc [25 mM] to the cultures is indicated with an asterisk. The area percentage were calculated using GraphPad. The areas are presented in percentage compound present in the culture. BHET is indicated in turquoise and MHET is indicated in magenta. Some impurities are present as a peak around 9.5 min retention time, this compound is called unknown and presented in purple. Individual data points are shown.1 B) Analysis of BHET degradation in agar plugs after 15 days of growth using LC-MS. The area percentage was calculated using GraphPad. *The agar plug of M145 was taken after 18 days. BHET is indicated in turquoise and MHET is indicated in magenta, TPA is represented in black. All error bars display the standard deviation and the individual datapoints are represented.*

Supplementary Figure 5

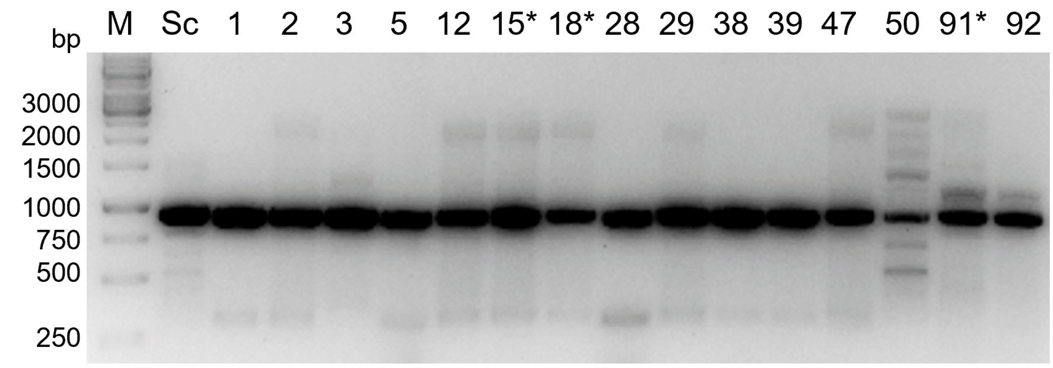

Figure 5: ***PCR of LipA gene in the active strains***

Supplementary Note 1

DNA sequence alignment of *lipA* variants. The cleavage site of the signal peptide was displayed with a magenta arrow.

```
MBT1    GTGCAGCAGAACCCCCACAC------------CCACGCGGCGCGTCCGGCGTTCCGTGGA  48
MBT2    GTGCAGCAGAACCCCCACAC------------CCACGCGGCGCGTCCGGCGTTCCGTGGA  48
MBT5    GTGCAGCAGAACCCCCACAC------------CCACGCGGCGCGTCCGGCGTTCCGTGGA  48
MBT12   GTGCAGCAGAACCCCCACAC------------CCACGCGGCGCGTCCGGCGTTCCGTGGA  48
MBT28   GTGCAGCAGAACCCCCACAC------------CCACGCGGCGCGTCCGGCGTTCCGTGGA  48
MBT29   GTGCAGCAGAACCCCCACAC------------CCACGCGGCGCGTCCGGCGTTCCGTGGA  48
MBT38   GTGCAGCAGAACCCCCACAC------------CCACGCGGCGCGTCCGGCGTTCCGTGGA  48
MBT39   GTGCAGCAGAACCCCCACAC------------CCACGCGGCGCGTCCGGCGTTCCGTGGA  48
MBT47   GTGCAGCAGAACCCCCACAC------------CCACGCGGCGCGTCCGGCGTTCCGTGGA  48
MBT50   GTGCAGCAGAACCCCCACAC------------CCACGCGGCGCGTCCGGCGTTCCGTGGA  48
MBT3    GTGCAGCAGAACCCCCACAC------------CCACGCGGCGCGTCCGGCGTTCCGTGGA  48
M145    GTGCAGCAGAACCCCCACACCCACGCCGCCCCAGGCGCCGCGCGCCCCGTCCTCCGGGGC  60
MBT92   GTGCAGCAGAACCCCCACACCCACGCGGCCCCCGGGGCCGCGCGCCCCGTCTTCCGGGGC  60

MBT1    CCCCGCCGGCGGCTCGCCGCTCTCACGGCCGCCGTGGCCGCCGCCGTCGCGCTCACCACC  108
MBT2    CCCCGCCGGCGGCTCGCCGCTCTCACGGCCGCCGTGGCCGCCGCCGTCGCGCTCACCACC  108
MBT5    CCCCGCCGGCGGCTCGCCGCTCTCACGGCCGCCGTGGCCGCCGCCGTCGCGCTCACCACC  108
MBT12   CCCCGCCGGCGGCTCGCCGCTCTCACGGCCGCCGTGGCCGCCGCCGTCGCGCTCACCACC  108
MBT28   CCCCGCCGGCGGCTCGCCGCTCTCACGGCCGCCGTGGCCGCCGCCGTCGCGCTCACCACC  108
MBT29   CCCCGCCGGCGGCTCGCCGCTCTCACGGCCGCCGTGGCCGCCGCCGTCGCGCTCACCACC  108
MBT38   CCCCGCCGGCGGCTCGCCGCTCTCACGGCCGCCGTGGCCGCCGCCGTCGCGCTCACCACC  108
MBT39   CCCCGCCGGCGGCTCGCCGCTCTCACGGCCGCCGTGGCCGCCGCCGTCGCGCTCACCACC  108
MBT47   CCCCGCCGGCGGCTCGCCGCTCTCACGGCCGCCGTGGCCGCCGCCGTCGCGCTCACCACC  108
MBT50   CCCCGCCGGCGGCTCGCCGCTCTCACGGCCGCCGTGGCCGCCGCCGTCGCGCTCACCACC  108
MBT3    CCCCGCCGGCGGCTCGCCGCTCTCACGGCCGCCGTGGCCGCCGCCGTCGCGCTCACCACC  108
M145    GTCCGGCGGCGGCTGGCCGCAGTGACGGCGGCCGTGGCCGCGGTCCTCGTGCTCGGCACC  120
MBT92   GTCCGGCGGCGGCTGGCCGGCGTGACGGCGGCCGTGGCCGCGGCCCTCGTGCTCGGCACC  120

MBT1    CTCACCGGCCCGGGCGCCCAGGCCGCCGACAACCCGTACGAGCGCGGCCCGGCGCCCACC  168
MBT2    CTCACCGGCCCGGGCGCCCAGGCCGCCGACAACCCGTACGAGCGCGGCCCGGCGCCCACC  168
MBT5    CTCACCGGCCCGGGCGCCCAGGCCGCCGACAACCCGTACGAGCGCGGCCCGGCGCCCACC  168
MBT12   CTCACCGGCCCGGGCGCCCAGGCCGCCGACAACCCGTACGAGCGCGGCCCGGCGCCCACC  168
MBT28   CTCACCGGCCCGGGCGCCCAGGCCGCCGACAACCCGTACGAGCGCGGCCCGGCGCCCACC  168
MBT29   CTCACCGGCCCGGGCGCCCAGGCCGCCGACAACCCGTACGAGCGCGGCCCGGCGCCCACC  168
MBT38   CTCACCGGCCCGGGCGCCCAGGCCGCCGACAACCCGTACGAGCGCGGCCCGGCGCCCACC  168
MBT39   CTCACCGGCCCGGGCGCCCAGGCCGCCGACAACCCGTACGAGCGCGGCCCGGCGCCCACC  168
MBT47   CTCACCGGCCCGGGCGCCCAGGCCGCCGACAACCCGTACGAGCGCGGCCCGGCGCCCACC  168
MBT50   CTCACCGGCCCGGGCGCCCAGGCCGCCGACAACCCGTACGAGCGCGGCCCGGCGCCCACC  168
MBT3    CTCACCGGCCCGGGCGCCCAGGCCGCCGACAACCCGTACGAGCGCGGCCCGGCGCCCACC  168
M145    CTCACCGGCCCGGGAGCCCAGGCCGCCGACAACCCGTACGAGCGCGGCCCGGCGCCCACC  180
MBT92   CTCACCGGCCCGGGCGCCCAGGCCGCGGACAACCCGTACGAGCGCGGCCCGGCGCCCACC  180

MBT1    GAGTCCAGCATCGAGGCCCTGCGCGGTCCGTACTCCGTGGCCGACACCAGCGTCTCCTCG  228
```

```
MBT2    GAGTCCAGCATCGAGGCCCTGCGCGGTCCGTACTCCGTGGCCGACACCAGCGTCTCCTCG   228
MBT5    GAGTCCAGCATCGAGGCCCTGCGCGGTCCGTACTCCGTGGCCGACACCAGCGTCTCCTCG   228
MBT12   GAGTCCAGCATCGAGGCCCTGCGCGGTCCGTACTCCGTGGCCGACACCAGCGTCTCCTCG   228
MBT28   GAGTCCAGCATCGAGGCCCTGCGCGGTCCGTACTCCGTGGCCGACACCAGCGTCTCCTCG   228
MBT29   GAGTCCAGCATCGAGGCCCTGCGCGGTCCGTACTCCGTGGCCGACACCAGCGTCTCCTCG   228
MBT38   GAGTCCAGCATCGAGGCCCTGCGCGGTCCGTACTCCGTGGCCGACACCAGCGTCTCCTCG   228
MBT39   GAGTCCAGCATCGAGGCCCTGCGCGGTCCGTACTCCGTGGCCGACACCAGCGTCTCCTCG   228
MBT47   GAGTCCAGCATCGAGGCCCTGCGCGGTCCGTACTCCGTGGCCGACACCAGCGTCTCCTCG   228
MBT50   GAGTCCAGCATCGAGGCCCTGCGCGGTCCGTACTCCGTGGCCGACACCAGCGTCTCCTCG   228
MBT3    GAGTCCAGCATCGAGGCCCTGCGCGGTCCGTACTCCGTGGCCGACACCAGCGTCTCCTCG   228
M145    GAGTCCAGCATCGAAGCCCTGCGCGGTCCCTACTCCGTGGCCGACACCAGCGTCTCCTCG   240
MBT92   GAGTCCAGCATCGAGGCCCTGCGCGGCCCCTACTCCGTGGCCGACACCAGGGTCTCCTCG   240

MBT1    CTCGCCGTCACCGGCTTCGGCGGCACCATCTACTACCCGACCAGCACCAGCGACGGC   288
MBT2    CTCGCCGTCACCGGCTTCGGCGGCACCATCTACTACCCGACCAGCACCAGCGACGGC   288
MBT5    CTCGCCGTCACCGGCTTCGGCGGCACCATCTACTACCCGACCAGCACCAGCGACGGC   288
MBT12   CTCGCCGTCACCGGCTTCGGCGGCACCATCTACTACCCGACCAGCACCAGCGACGGC   288
MBT28   CTCGCCGTCACCGGCTTCGGCGGCACCATCTACTACCCGACCAGCACCAGCGACGGC   288
MBT29   CTCGCCGTCACCGGCTTCGGCGGCACCATCTACTACCCGACCAGCACCAGCGACGGC   288
MBT38   CTCGCCGTCACCGGCTTCGGCGGCACCATCTACTACCCGACCAGCACCAGCGACGGC   288
MBT39   CTCGCCGTCACCGGCTTCGGCGGCACCATCTACTACCCGACCAGCACCAGCGACGGC   288
MBT47   CTCGCCGTCACCGGCTTCGGCGGCACCATCTACTACCCGACCAGCACCAGCGACGGC   288
MBT50   CTCGCCGTCACCGGCTTCGGCGGCACCATCTACTACCCGACCAGCACCAGCGACGGC   288
MBT3    CTCGCCGTCACCGGCTTCGGCGGCACCATCTACTACCCGACCAGCACCAGCGACGGC   288
M145    CTCGCGGTCACCGGATTCGGCGGCACCATCTACTACCCGACCAGCACCAGCGACGGC   300
MBT92   CTCGCGGTCACCGGATTCGGCGGCACCATCTACTACCCGACCAGCACCAGCGACGGC   300

MBT1    ACCTTCGGCGCCGTGGTGATCTCGCCCGGCTTCACCGCGTACCAGTCGTCCATCGCCTGG   348
MBT2    ACCTTCGGCGCCGTGGTGATCTCGCCCGGCTTCACCGCGTACCAGTCGTCCATCGCCTGG   348
MBT5    ACCTTCGGCGCCGTGGTGATCTCGCCCGGCTTCACCGCGTACCAGTCGTCCATCGCCTGG   348
MBT12   ACCTTCGGCGCCGTGGTGATCTCGCCCGGCTTCACCGCGTACCAGTCGTCCATCGCCTGG   348
MBT28   ACCTTCGGCGCCGTGGTGATCTCGCCCGGCTTCACCGCGTACCAGTCGTCCATCGCCTGG   348
MBT29   ACCTTCGGCGCCGTGGTGATCTCGCCCGGCTTCACCGCGTACCAGTCGTCCATCGCCTGG   348
MBT38   ACCTTCGGCGCCGTGGTGATCTCGCCCGGCTTCACCGCGTACCAGTCGTCCATCGCCTGG   348
MBT39   ACCTTCGGCGCCGTGGTGATCTCGCCCGGCTTCACCGCGTACCAGTCGTCCATCGCCTGG   348
MBT47   ACCTTCGGCGCCGTGGTGATCTCGCCCGGCTTCACCGCGTACCAGTCGTCCATCGCCTGG   348
MBT50   ACCTTCGGCGCCGTGGTGATCTCGCCCGGCTTCACCGCGTACCAGTCGTCCATCGCCTGG   348
MBT3    ACCTTCGGCGCCGTGGTGATCTCGCCCGGCTTCACCGCGTACCAGTCGTCCATCGCCTGG   348
M145    ACGTTCGGCGCCGTCGTCATCGCGCCCGGGTTCACCGCGTACCAGTCGTCCATCGCCTGG   360
MBT92   ACGTTCGGCGCCGTGGCGATCGCACCCGGCTTCACCGCGTACCAGTCCTCCATGGCCTGG   360

MBT1    CTCGGTCCGCGGCTGGCCTCGCAGGGCTTCGTGGTCTTCACCATCGACACCAACACCACG   408
MBT2    CTCGGTCCGCGGCTGGCCTCGCAGGGCTTCGTGGTCTTCACCATCGACACCAACACCACG   408
MBT5    CTCGGTCCGCGGCTGGCCTCGCAGGGCTTCGTGGTCTTCACCATCGACACCAACACCACG   408
MBT12   CTCGGTCCGCGGCTGGCCTCGCAGGGCTTCGTGGTCTTCACCATCGACACCAACACCACG   408
MBT28   CTCGGTCCGCGGCTGGCCTCGCAGGGCTTCGTGGTCTTCACCATCGACACCAACACCACG   408
MBT29   CTCGGTCCGCGGCTGGCCTCGCAGGGCTTCGTGGTCTTCACCATCGACACCAACACCACG   408
MBT38   CTCGGTCCGCGGCTGGCCTCGCAGGGCTTCGTGGTCTTCACCATCGACACCAACACCACG   408
MBT39   CTCGGTCCGCGGCTGGCCTCGCAGGGCTTCGTGGTCTTCACCATCGACACCAACACCACG   408
```

```
MBT47   CTCGGTCCGCGGCTGGCCTCGCAGGGCTTCGTGGTCTTCACCATCGACACCAACACCACG   408
MBT50   CTCGGTCCGCGGCTGGCCTCGCAGGGCTTCGTGGTCTTCACCATCGACACCAACACCACG   408
MBT3    CTCGGTCCGCGGCTGGCCTCGCAGGGCTTCGTGGTCTTCACCATCGACACCAACACCACG   408
M145    CTCGGCCCGCGCCTGGCCTCCCAGGGCTTCGTGGTCTTCACCATCGACACCAACACCACG   420
MBT92   CTCGGTCCGCGGCTGGCCTCCCAGGGCTTCGTGGTCTTCACCATCGACACCAACACCACG   420

MBT1    CTGGACCAGCCCGACTCCCGAGGCCGGCAACTGCTGGCCGCCCTGGACTACCTGACCGAG   468
MBT2    CTGGACCAGCCCGACTCCCGAGGCCGGCAACTGCTGGCCGCCCTGGACTACCTGACCGAG   468
MBT5    CTGGACCAGCCCGACTCCCGAGGCCGGCAACTGCTGGCCGCCCTGGACTACCTGACCGAG   468
MBT12   CTGGACCAGCCCGACTCCCGAGGCCGGCAACTGCTGGCCGCCCTGGACTACCTGACCGAG   468
MBT28   CTGGACCAGCCCGACTCCCGAGGCCGGCAACTGCTGGCCGCCCTGGACTACCTGACCGAG   468
MBT29   CTGGACCAGCCCGACTCCCGAGGCCGGCAACTGCTGGCCGCCCTGGACTACCTGACCGAG   468
MBT38   CTGGACCAGCCCGACTCCCGAGGCCGGCAACTGCTGGCCGCCCTGGACTACCTGACCGAG   468
MBT39   CTGGACCAGCCCGACTCCCGAGGCCGGCAACTGCTGGCCGCCCTGGACTACCTGACCGAG   468
MBT47   CTGGACCAGCCCGACTCCCGAGGCCGGCAACTGCTGGCCGCCCTGGACTACCTGACCGAG   468
MBT50   CTGGACCAGCCCGACTCCCGAGGCCGGCAACTGCTGGCCGCCCTGGACTACCTGACCGAG   468
MBT3    CTGGACCAGCCCGACTCCCGAGGCCGGCAACTGCTGGCCGCCCTGGACTACCTGACCGAG   468
M145    CTGGACCAGCCCGACTCCCGCGGGCGGCAACTGCTGGCCGCCCTGGACTACCTGACCGGG   480
MBT92   CTGGACCAGCCCGACTCCCGCGGCCGCCAACTGCTGGCCGCCCTGGACTACCTCACCGAG   480

MBT1    CGCAGCTCCGTCCGGGGACGGATCGACAGCAGCCGGCTCGGCGTCATGGGCCACTCCATG   528
MBT2    CGCAGCTCCGTCCGGGGACGGATCGACAGCAGCCGGCTCGGCGTCATGGGCCACTCCATG   528
MBT5    CGCAGCTCCGTCCGGGGACGGATCGACAGCAGCCGGCTCGGCGTCATGGGCCACTCCATG   528
MBT12   CGCAGCTCCGTCCGGGGACGGATCGACAGCAGCCGGCTCGGCGTCATGGGCCACTCCATG   528
MBT28   CGCAGCTCCGTCCGGGGACGGATCGACAGCAGCCGGCTCGGCGTCATGGGCCACTCCATG   528
MBT29   CGCAGCTCCGTCCGGGGACGGATCGACAGCAGCCGGCTCGGCGTCATGGGCCACTCCATG   528
MBT38   CGCAGCTCCGTCCGGGGACGGATCGACAGCAGCCGGCTCGGCGTCATGGGCCACTCCATG   528
MBT39   CGCAGCTCCGTCCGGGGACGGATCGACAGCAGCCGGCTCGGCGTCATGGGCCACTCCATG   528
MBT47   CGCAGCTCCGTCCGGGGACGGATCGACAGCAGCCGGCTCGGCGTCATGGGCCACTCCATG   528
MBT50   CGCAGCTCCGTCCGGGGACGGATCGACAGCAGCCGGCTCGGCGTCATGGGCCACTCCATG   528
MBT3    CGCAGCTCCGTCCGGGGACGGATCGACAGCAGCCGGCTCGGCGTCATGGGCCACTCCATG   528
M145    CGCAGCTCCGTCCGCGGCCGGATCGACAGCGGCCGACTCGGCGTCATGGGCCACTCCATG   540
MBT92   CGCAGCTCCGTCCGCGGCCGGATCGACAGCAGCCGCCTCGGCGTGATGGGCCACTCCATG   540

MBT1    GGCGGCGGAGGCTCGCTGGAGGCCGCCAAGTCCCGTCCGTCGCTCCAGGCGGCGATCCCG   588
MBT2    GGCGGCGGAGGCTCGCTGGAGGCCGCCAAGTCCCGTCCGTCGCTCCAGGCGGCGATCCCG   588
MBT5    GGCGGCGGAGGCTCGCTGGAGGCCGCCAAGTCCCGTCCGTCGCTCCAGGCGGCGATCCCG   588
MBT12   GGCGGCGGAGGCTCGCTGGAGGCCGCCAAGTCCCGTCCGTCGCTCCAGGCGGCGATCCCG   588
MBT28   GGCGGCGGAGGCTCGCTGGAGGCCGCCAAGTCCCGTCCGTCGCTCCAGGCGGCGATCCCG   588
MBT29   GGCGGCGGAGGCTCGCTGGAGGCCGCCAAGTCCCGTCCGTCGCTCCAGGCGGCGATCCCG   588
MBT38   GGCGGCGGAGGCTCGCTGGAGGCCGCCAAGTCCCGTCCGTCGCTCCAGGCGGCGATCCCG   588
MBT39   GGCGGCGGAGGCTCGCTGGAGGCCGCCAAGTCCCGTCCGTCGCTCCAGGCGGCGATCCCG   588
MBT47   GGCGGCGGAGGCTCGCTGGAGGCCGCCAAGTCCCGTCCGTCGCTCCAGGCGGCGATCCCG   588
MBT50   GGCGGCGGAGGCTCGCTGGAGGCCGCCAAGTCCCGTCCGTCGCTCCAGGCGGCGATCCCG   588
MBT3    GGCGGCGGAGGCTCGCTGGAGGCCGCCAAGTCCCGTCCGTCGCTCCAGGCGGCGATCCCG   588
M145    GGCGGCGGCGGCACCCTGGAGGCCGCCAAGTCCCGGCCGTCGCTCCAGGCGGCGATCCCC   600
MBT92   GGCGGCGGCGGCACTTTGGAGGCCGCCAAGTCCCGGCCGTCCCTCCAGGCGGCGATCCCC   600
```

MBT1	CTCACCCCCTGGAACCTGGACAAGAGCTGGCCGGAGGTCAGCACCCCGACCCTGATCGTG	648
MBT2	CTCACCCCCTGGAACCTGGACAAGAGCTGGCCGGAGGTCAGCACCCCGACCCTGATCGTG	648
MBT5	CTCACCCCCTGGAACCTGGACAAGAGCTGGCCGGAGGTCAGCACCCCGACCCTGATCGTG	648
MBT12	CTCACCCCCTGGAACCTGGACAAGAGCTGGCCGGAGGTCAGCACCCCGACCCTGATCGTG	648
MBT28	CTCACCCCCTGGAACCTGGACAAGAGCTGGCCGGAGGTCAGCACCCCGACCCTGATCGTG	648
MBT29	CTCACCCCCTGGAACCTGGACAAGAGCTGGCCGGAGGTCAGCACCCCGACCCTGATCGTG	648
MBT38	CTCACCCCCTGGAACCTGGACAAGAGCTGGCCGGAGGTCAGCACCCCGACCCTGATCGTG	648
MBT39	CTCACCCCCTGGAACCTGGACAAGAGCTGGCCGGAGGTCAGCACCCCGACCCTGATCGTG	648
MBT47	CTCACCCCCTGGAACCTGGACAAGAGCTGGCCGGAGGTCAGCACCCCGACCCTGATCGTG	648
MBT50	CTCACCCCCTGGAACCTGGACAAGAGCTGGCCGGAGGTCAGCACCCCGACCCTGATCGTG	648
MBT3	CTCACCCCCTGGAACCTGGACAAGAGCTGGCCGGAGGTCAGCACCCCGACCCTGATCGTG	648
M145	CTCACCCCCTGGAACCTGGACAAGAGCTGGCCGGAGGTCAGCACGCCGACGCTGGTCGTC	660
MBT92	CTCACGCCCTGGAACCTGGACAAGAGCTGGCCCGAGGTCAGCACCCCGACGCTGATCGTG	660

MBT1	GGGGCCGACGGCGACACGGTCGCGCCCGTCTCCTCGCACTCCGAGCCTTTCTACTCCAGC	708
MBT2	GGGGCCGACGGCGACACGGTCGCGCCCGTCTCCTCGCACTCCGAGCCTTTCTACTCCAGC	708
MBT5	GGGGCCGACGGCGACACGGTCGCGCCCGTCTCCTCGCACTCCGAGCCTTTCTACTCCAGC	708
MBT12	GGGGCCGACGGCGACACGGTCGCGCCCGTCTCCTCGCACTCCGAGCCTTTCTACTCCAGC	708
MBT28	GGGGCCGACGGCGACACGGTCGCGCCCGTCTCCTCGCACTCCGAGCCTTTCTACTCCAGC	708
MBT29	GGGGCCGACGGCGACACGGTCGCGCCCGTCTCCTCGCACTCCGAGCCTTTCTACTCCAGC	708
MBT38	GGGGCCGACGGCGACACGGTCGCGCCCGTCTCCTCGCACTCCGAGCCTTTCTACTCCAGC	708
MBT39	GGGGCCGACGGCGACACGGTCGCGCCCGTCTCCTCGCACTCCGAGCCTTTCTACTCCAGC	708
MBT47	GGGGCCGACGGCGACACGGTCGCGCCCGTCTCCTCGCACTCCGAGCCTTTCTACTCCAGC	708
MBT50	GGGGCCGACGGCGACACGGTCGCGCCCGTCTCCTCGCACTCCGAGCCTTTCTACTCCAGC	708
MBT3	GGTGCCGACGGCGACACGGTCGCGCCCGTCTCCTCGCACTCCGAGCCGTTCTACTCCAGC	708
M145	GGGGCCGACGGCGACACGATCGCCCCCGTGGCCTCGCACGCCGAACCGTTCTACTCCGGC	720
MBT92	GGGGCCGACGGCGACACGATCGCCCCCGTGGCCTCGCACGCCGAGCCGTTCTACTCCGGC	720

MBT1	CTGCCGTCCGGAACGGACCGCGCCTACCTGGAGCTGAACAACGCGACCCACTTCTCGCCG	768
MBT2	CTGCCGTCCGGAACGGACCGCGCCTACCTGGAGCTGAACAACGCGACCCACTTCTCGCCG	768
MBT5	CTGCCGTCCGGAACGGACCGCGCCTACCTGGAGCTGAACAACGCGACCCACTTCTCGCCG	768
MBT12	CTGCCGTCCGGAACGGACCGCGCCTACCTGGAGCTGAACAACGCGACCCACTTCTCGCCG	768
MBT28	CTGCCGTCCGGAACGGACCGCGCCTACCTGGAGCTGAACAACGCGACCCACTTCTCGCCG	768
MBT29	CTGCCGTCCGGAACGGACCGCGCCTACCTGGAGCTGAACAACGCGACCCACTTCTCGCCG	768
MBT38	CTGCCGTCCGGAACGGACCGCGCCTACCTGGAGCTGAACAACGCGACCCACTTCTCGCCG	768
MBT39	CTGCCGTCCGGAACGGACCGCGCCTACCTGGAGCTGAACAACGCGACCCACTTCTCGCCG	768
MBT47	CTGCCGTCCGGAACGGACCGCGCCTACCTGGAGCTGAACAACGCGACCCACTTCTCGCCG	768
MBT50	CTGCCGTCCGGAACGGACCGCGCCTACCTGGAGCTGAACAACGCGACCCACTTCTCGCCG	768
MBT3	CTGCCGTCCGGAACGGACCGCGCCTACCTGGAGCTGAACAACGCGACCCACTTCTCGCCG	768
M145	CTGCCCTCGTCGACCGACCGGGCCTATCTGGAGCTGAACAACGCGACCCACTTCTCGCCC	780
MBT92	CTGCCCTCCGCGACCGACCGGGCCTATCTGGAGCTGAACGGCGCGACGCACTTCTCGCCC	780

MBT1	AACACGTCGAACACCACGATCGCGAAGTACAGCATCTCCTGGCTCAAGCGGTTCATCGAC	828
MBT2	AACACGTCGAACACCACGATCGCGAAGTACAGCATCTCCTGGCTCAAGCGGTTCATCGAC	828
MBT5	AACACGTCGAACACCACGATCGCGAAGTACAGCATCTCCTGGCTCAAGCGGTTCATCGAC	828
MBT12	AACACGTCGAACACCACGATCGCGAAGTACAGCATCTCCTGGCTCAAGCGGTTCATCGAC	828
MBT28	AACACGTCGAACACCACGATCGCGAAGTACAGCATCTCCTGGCTCAAGCGGTTCATCGAC	828
MBT29	AACACGTCGAACACCACGATCGCGAAGTACAGCATCTCCTGGCTCAAGCGGTTCATCGAC	828

```
MBT38   AACACGTCGAACACCACGATCGCGAAGTACAGCATCTCCTGGCTCAAGCGGTTCATCGAC   828
MBT39   AACACGTCGAACACCACGATCGCGAAGTACAGCATCTCCTGGCTCAAGCGGTTCATCGAC   828
MBT47   AACACGTCGAACACCACGATCGCGAAGTACAGCATCTCCTGGCTCAAGCGGTTCATCGAC   828
MBT50   AACACGTCGAACACCACGATCGCGAAGTACAGCATCTCCTGGCTCAAGCGGTTCATCGAC   828
MBT3    AACACGTCGAACACCACGATCGCGAAGTACAGCATCTCCTGGCTCAAGCGGTTCATCGAC   828
M145    AACACGTCCAACACGACGATCGCGAAGTACAGCATCTCCTGGCTCAAGCGCTTCATCGAC   840
MBT92   AACTCGTCCAACACGACGATCGCGAAGTACAGCGTCTCCTGGCTCAAGCGGTTCATCGAC   840

MBT1    AACGACACCCGCTACGAGCAGTTCCTGTGCCCGCTGCCCCGGCCGAGCCTGACCATCGAG   888
MBT2    AACGACACCCGCTACGAGCAGTTCCTGTGCCCGCTGCCCCGGCCGAGCCTGACCATCGAG   888
MBT5    AACGACACCCGCTACGAGCAGTTCCTGTGCCCGCTGCCCCGGCCGAGCCTGACCATCGAG   888
MBT12   AACGACACCCGCTACGAGCAGTTCCTGTGCCCGCTGCCCCGGCCGAGCCTGACCATCGAG   888
MBT28   AACGACACCCGCTACGAGCAGTTCCTGTGCCCGCTGCCCCGGCCGAGCCTGACCATCGAG   888
MBT29   AACGACACCCGCTACGAGCAGTTCCTGTGCCCGCTGCCCCGGCCGAGCCTGACCATCGAG   888
MBT38   AACGACACCCGCTACGAGCAGTTCCTGTGCCCGCTGCCCCGGCCGAGCCTGACCATCGAG   888
MBT39   AACGACACCCGCTACGAGCAGTTCCTGTGCCCGCTGCCCCGGCCGAGCCTGACCATCGAG   888
MBT47   AACGACACCCGCTACGAGCAGTTCCTGTGCCCGCTGCCCCGGCCGAGCCTGACCATCGAG   888
MBT50   AACGACACCCGCTACGAGCAGTTCCTGTGCCCGCTGCCCCGGCCGAGCCTGACCATCGAG   888
MBT3    AACGACACCCGCTACGAGCAGTTCCTGTGCCCGCTGCCCCGGCCGAGCCTGACCATCGAG   888
M145    GACGACACCCGCTACGAGCAGTTCCTGTGCCCGCTGCCGCGGCCGAGCCTGACGATCGAG   900
MBT92   AACGACACCCGCTACGAGCAGTTCCTGTGCCCGCTGCCGCGGCCGAGTCTGACGATCGAG   900

MBT1    GAGTACCGGGGCAACTGCCCGCACGGCTCCTGA921
MBT2    GAGTACCGGGGCAACTGCCCGCACGGCTCCTGA921
MBT5    GAGTACCGGGGCAACTGCCCGCACGGCTCCTGA921
MBT12   GAGTACCGGGGCAACTGCCCGCACGGCTCCTGA      921
MBT28   GAGTACCGGGGCAACTGCCCGCACGGCTCCTGA      921
MBT29   GAGTACCGGGGCAACTGCCCGCACGGCTCCTGA      921
MBT38   GAGTACCGGGGCAACTGCCCGCACGGCTCCTGA      921
MBT39   GAGTACCGGGGCAACTGCCCGCACGGCTCCTGA      921
MBT47   GAGTACCGGGGCAACTGCCCGCACGGCTCCTGA      921
MBT50   GAGTACCGGGGCAACTGCCCGCACGGCTCCTGA      921
MBT3    GAGTACCGGGGCAACTGCCCGCACGGCTCCTGA921
M145    GAGTACCGGGGCAACTGCCCGCACGGTTCCTGA      933
MBT92   GAGTACCGGGGCAACTGCCCGCACGGCTCCTGA      933
```

Supplementary Figure 6

```
MBT1,2,3,5,12,28,29,38,39,47,50   MQQNPHTHA----ARPAFRGPRRRLAALTAAVAAAVALTTLTGPGAQAADNPYERGPAPT   56
M145                              MQQNPHTHAAPGAARPVLRGVRRRLAAVTAAVAAVLVLGTLTGPGAQAADNPYERGPAPT   60
MBT92                             MQQNPHTHAAPGAARPVFRGVRRRLAGVTAAVAAALVLGTLTGPGAQAADNPYERGPAPT   60
                                           ****    *  *     **      *** *

MBT1,2,3,5,12,28,29,38,39,47,50   ESSIEALRGPYSVADTSVSSLAVTGFGGGTIYYPTSTSDGTFGAVVISPGFTAYQSSIAW   116
M145                              ESSIEALRGPYSVADTSVSSLAVTGFGGGTIYYPTSTSDGTFGAVVIAPGFTAYQSSIAW   120
MBT92                             ESSIEALRGPYSVADTRVSSLAVTGFGGGTIYYPTSTSDGTFGAVAIAPGFTAYQSSMAW   120
                                                  *                            *           *

MBT1,2,3,5,12,28,29,38,39,47,50   LGPRLASQGFVVFTIDTNTTLDQPDSRGRQLLAALDYLTERSSVRGRIDSSRLGVMGHSM   176
M145                              LGPRLASQGFVVFTIDTNTTLDQPDSRGRQLLAALDYLTGRSSVRGRIDSGRLGVMGHSM   180
MBT92                             LGPRLASQGFVVFTIDTNTTLDQPDSRGRQLLAALDYLTERSSVRGRIDSSRLGVMGHSM   180
                                                                         *          *

MBT1,2,3,5,12,28,29,38,39,47,50   GGGGSLEAAKSRPSLQAAIPLTPWNLDKSWPEVSTPTLIVGADGDTVAPVSSHSEPFYSS   236
M145                              GGGGTLEAAKSRPSLQAAIPLTPWNLDKSWPEVSTPTLVVGADGDTIAPVASHAEPFYSG   240
MBT92                             GGGGTLEAAKSRPSLQAAIPLTPWNLDKSWPEVSTPTLIVGADGDTIAPVASHAEPFYSG   240
                                      *                                 *       *   *  *     *

MBT1,2,3,5,12,28,29,38,39,47,50   LPSGTDRAYLELNNATHFSPNTSNTTIAKYSISWLKRFIDNDTRYEQFLCPLPRPSLTIE   296
M145                              LPSSTDRAYLELNNATHFSPNTSNTTIAKYSISWLKRFIDDDTRYEQFLCPLPRPSLTIE   300
MBT92                             LPSATDRAYLELNGATHFSPNSSNTTIAKYSVSWLKRFIDNDTRYEQFLCPLPRPSLTIE   300
                                     *         *       *         *        *

MBT1,2,3,5,12,28,29,38,39,47,50   EYRGNCPHGS*      306
M145                              EYRGNCPHGS*      310
MBT92                             EYRGNCPHGS*      310
```

Figure 6: ***Protein alignment of the LipA variants.***
The divergent sequence of the signal peptide is visualized in cyan. The cleavage site of the signal peptide was displayed with a magenta arrow. Divergent amino acids are displayed in magenta.

Supplementary Figure 7

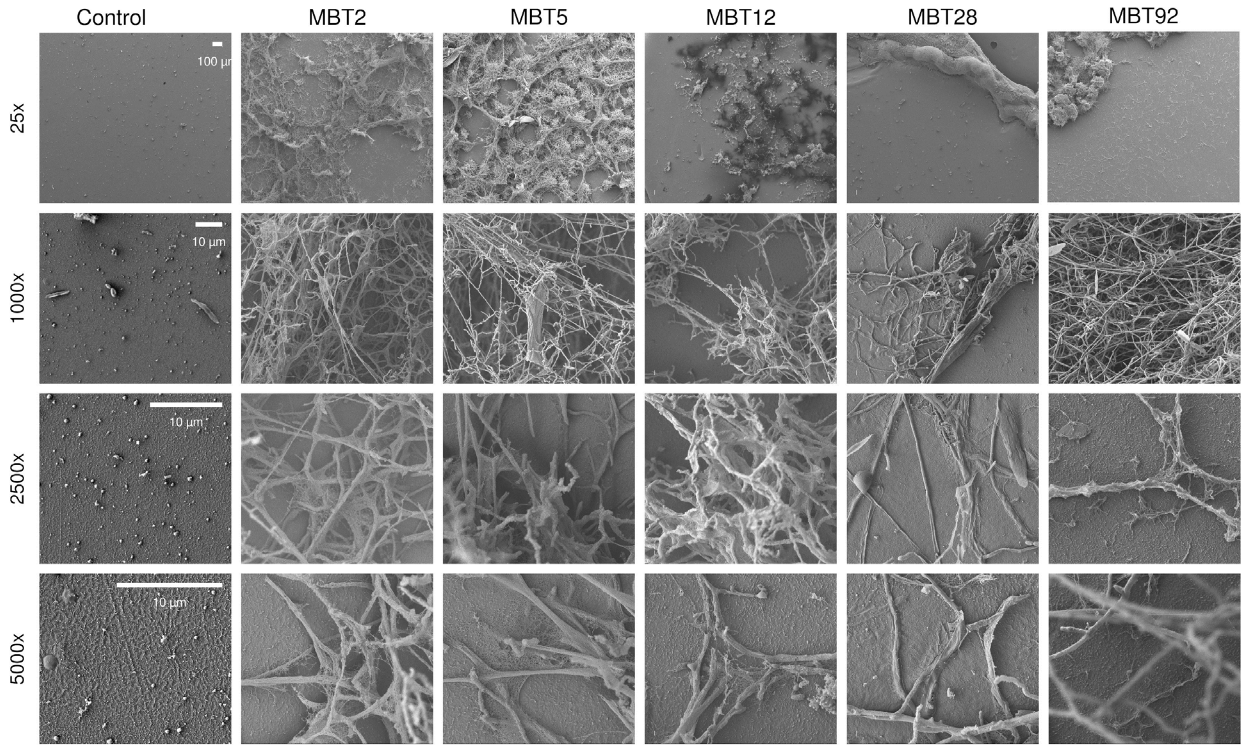

Figure 7: ***Scanning electron micrographs of MBT2, 5, 12, 28 and 92 on amorphous PET film.***
PET films incubated for 2 weeks at 30 °C in NMM with 0.05 % [w/v] glucose with 10^7 spores and inoculated for 2 weeks at 30 °C. From left to right: control, strain MBT2, MBT5, MBT12, MBT28 and MBT92. From top to bottom the magnifications 25 x, 1000 x, 2500 x and 5000x.

Supplementary Note 2

A LipA knock-out was created via CRISPR-Cas9 using homology-directed repair. Two homologous arms of approximately 1000bp with a 40nt overlap at both sides of LipA have been amplified via PCR on genomic DNA of M145. These homologous arms were cloned into the pCRISPomyces-2 via three-piece Gibson Assembly using a XbaI site. Additionally, a sgRNA was annealed into the plasmid using Golden Gate cloning. Primers for these homologous arms bordering LipA and sgRNA were made using the protocol of Cobb and colleagues. Assembled plasmids were confirmed via sequencing.

Supplementary Figure 8

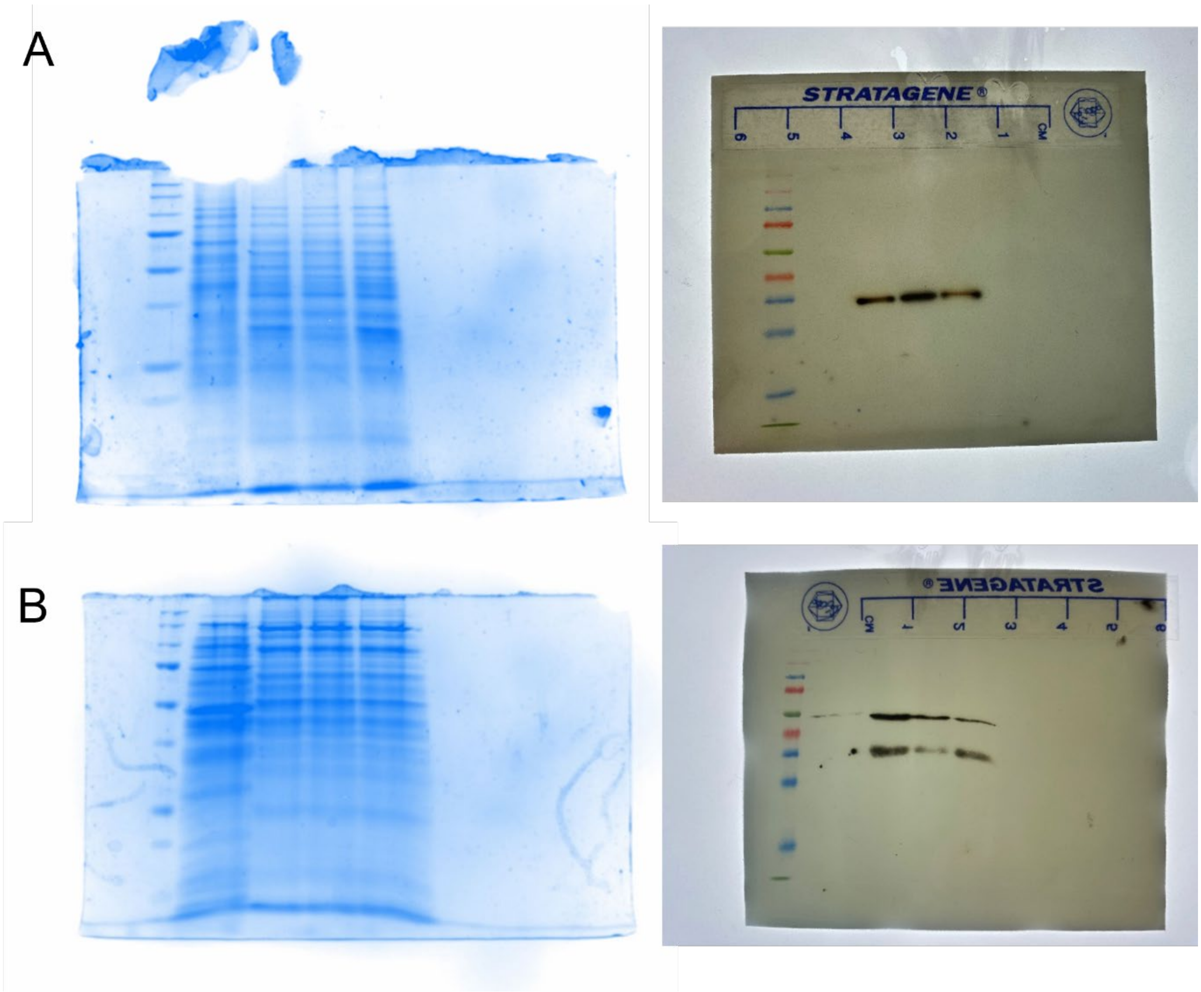

Figure 8: ***Original images of SDS-PAGE and Western blot of expression of the LipA variants in S. coelicolor M145 ΔlipA in NMM and TSBS medium.***

A) Raw image of SDS-PAGE and Western Blot of concentrated samples on NMM medium, faint band around ~32 kDa in the overexpression strains, this band is not present in wild-type strain M145. B) Raw image of SDS-PAGE of concentrated samples on TSBS medium, a faint band around ~32 kDa was observed in the overexpression strains this band was not present in wild-type strain M145.

Supplementary Figure 9

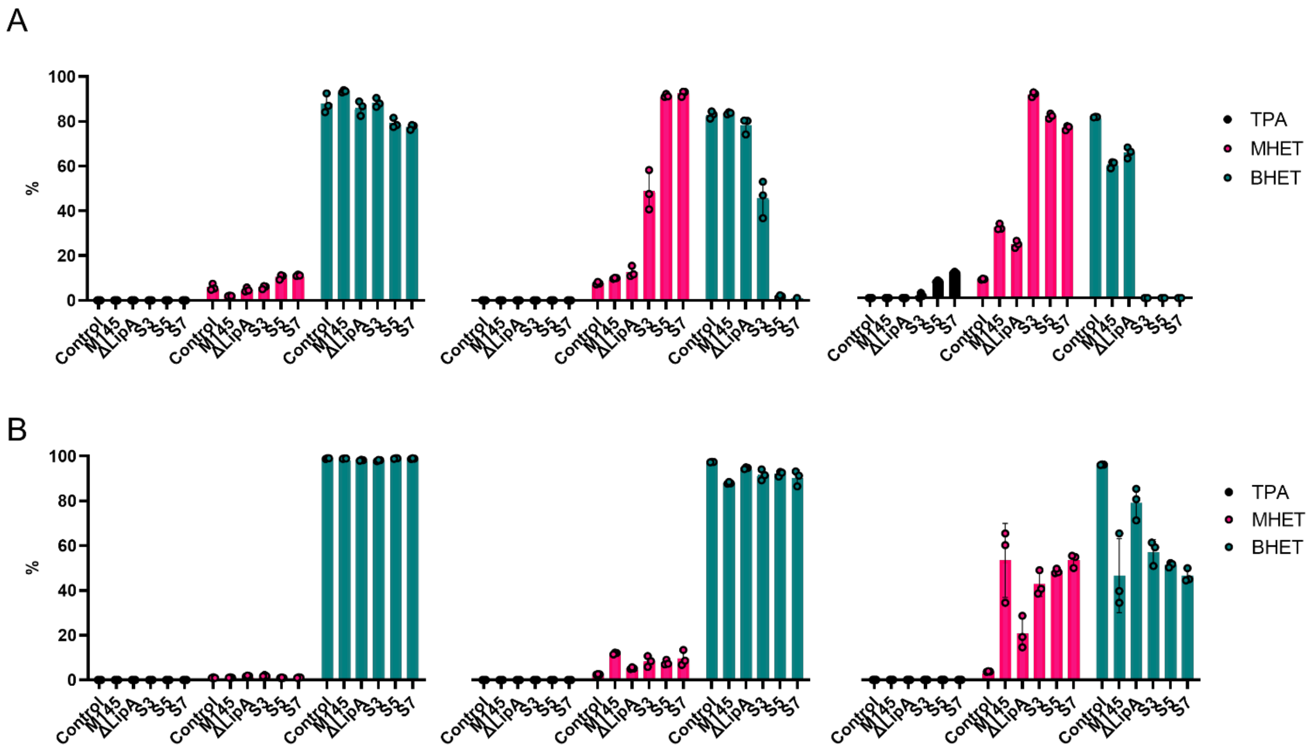

Figure 9: ***Graphs displaying individual measurements of BHET degradation of overexpression strains*** *A) Analysis of BHET degradation in NMM of the wild-type strain, ΔlipA, S3, S5 and S7 using LC-MS (n=3). Samples were taken at 24 h, 48 h and 72 h. The percentage BHET is presented in turquoise, the percentage MHET in magenta and the percentage TPA in black. The area percentage was calculated using GraphPad.*

B) Analysis of BHET degradation in TSBS of the wild-type strain, ΔlipA, S3, S5 and S7 using LC-MS. Samples were taken at 24 h, 48 h and 72 h. The percentage BHET is presented in turquoise, the percentage MHET in magenta and the percentage TPA in black. the error bars display the standard deviation. Individual datapoints are displayed in the figure.

Supplementary Figure 10

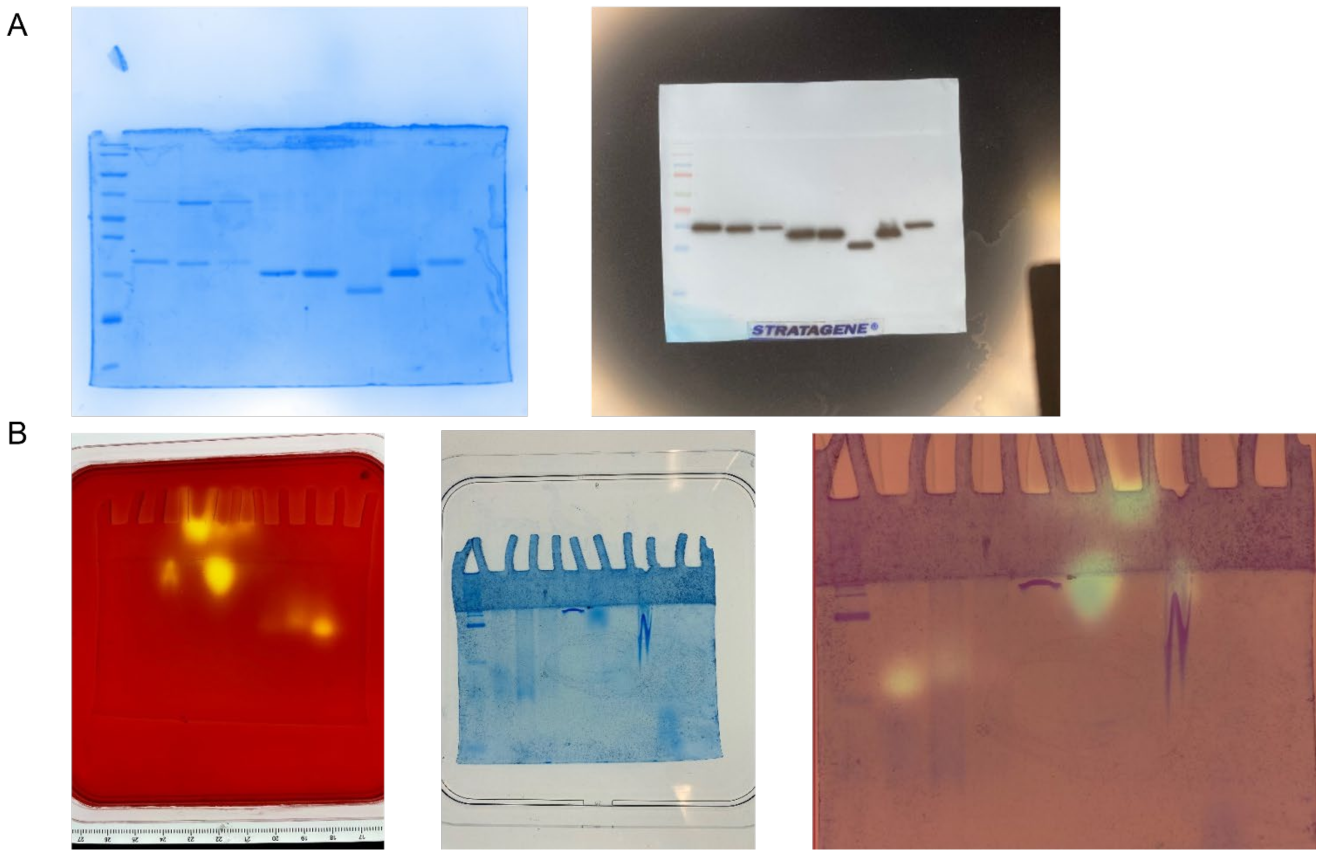

Figure 10: ***Raw gel images of*** **expression, purification and a zymogram of Lipase A enzymes in** ***E. coli.***

A) Original SDS-PAGE and Western blot of the purified Lipase A variants, along with the PET40 (40), TfCut2, LCC, IsPETase (PET) and PET46 (46); 1: Ladder, mutlicolor broad range protein ladder; 2: S2LipA sample (30kDa) ; 3: S92LipA (30kDa); 4: ScLipA (30kDa), 5: PET40 (28kDa), 6: TfCut2 (28kDa), 7: LCC (27kDa), IsPETase (27kDa), 8: PET46 (30kDa). The Lipase variants show two bands one around 60 kDa and one around 35kDa. C) Original Zymogram, Native SDS-PAGE and overlay of both of 750 ng of purified enzymes and LipA variants on 1% tributyrin. The Lipase A variants, PET40, TfCut2, LCC and PETase show degradation of tributyrin (yellow spots). NativeMarker used as marker and visualised by overlaying Native SDS-PAGE with zymogram (image on the right).

Supplementary Table 3

Table 3: ***Medium compositions for screening on plate***

Experiment	Medium	Agar	Addition
Toxicity screen	StrepMM	Iberian Agar	Mannitol 25 mM + BHET 0, 10 mM, 20 mM, 30 mM and 40 mM
Bulk screens	StrepMM	Iberian Agar<br>Difco agar<br>Agar-Agar	+/- BHET 10 mM<br>+/- GlcNAc 25 mM
Individual screens<br>*S. coelicolor* and active strains	StrepMM	Difco Agar	+/- Mannitol 25 mM<br>+/- BHET 10 mM<br>+/- GlcNAc 25 mM

Supplementary Table 4

*Table 4: **Primers for PCR and sequencing LipA variants***

Primer name	Sequence	Purpose	source
JA_G_F1_MBT2/12_SCO0713	GCGTCAGGAGCCGTGCG	PCR LipA	This work
JA_R1_MBT2/12_SCO0713	GTGCAGCAGAACCCCCACAC	PCR LipA	This work
pJET2.1_F	CGACTCACTATAGGGAGAGCGGC	pJET2.1 Sequencing	Thermofisher CloneJET PCR Kit K1231
pJET2.1_R	AAGAACATCGATTTTCCATGGCAG	pJET2.1 Sequencing	Thermofisher CloneJET PCR Kit K1231

Supplementary Table 5

*Table 5: **sgRNA and primers for homologous arms, diagnostic PCR and sequencing***

Name	Sequence	Length (nt)	Purpose
sgRNA1-F	ACGCTCCAGCATCGAAGCCCTGCG	24	sgRNA
sgRNA1-R	AAACCGCAGGGCTTCGATGCTGGA	24	sgRNA
JA_MC_F_HA1	TGCCGCCGGGCGTTTTTTATGGTCACCGGCCAGGACGA	38	Amplification Homologous Arm
JA_MC_F_HA2	CGTGCGGGCAGGTGTGGGGGTTCTGCTG	28	Amplification Homologous Arm
JA_MC_R_HA1	CCCCCACACCTGCCCGCACGGTTCCTGA	28	Amplification Homologous Arm
JA_MC_R_HA2*	CTTTTTACGGTTCCTGGCCTCGTGAGGCTGAGCGTGAG	38	Amplification Homologous Arm
JA_MRJ_F_KO_SCO0713	GTGCACCGTTCGACGGACGA	20	Diagnostic PCR and forward sequencing
JA_R1_MBT2/12_SCO0713	GTGCAGCAGAACCCCCACAC	20	Diagnostic PCR and reverse sequencing

Supplementary Figure 11

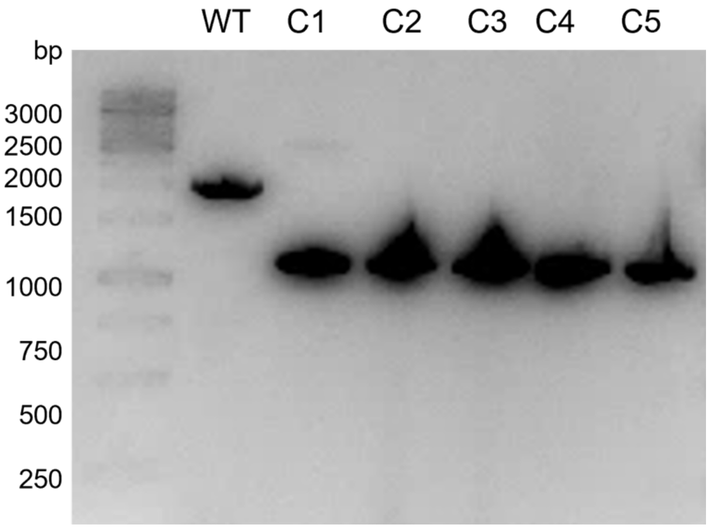

Figure 11: ***Diagnostic PCR of the WT and 5 knock-out colonies***
Expected size WT is 2000bp (HA1+LipA), Expected size knock-out 1000bp (HA1).

Supplementary Table 6

*Table 6: **Primers for construction and sequencing of pSET152_XLipA***

Name	Sequence
JA_MC_SCO0713_pSET_F	CTGTAGCTTACATATGCAGCAGAACCCCCACAC
JA_MC_SCO0713_145_92_pSET_R	ATCGAGGATCCTTCAGTGGTGGTGGTGGTGGTGGGAACCGTGCGGGCAGTT
JA_MC_SCO0713_2_pSET_R	ATCGAGGATCCTTCAGTGGTGGTGGTGGTGGTGGGAGCCGTGCGGGCAGTT
JA_M13_F (sequencing)	caggaaacagctatgacatgat
JA_M13_R (sequencing)	gtaaaacgacggccagt

NdeI site is marked in RED
BamHI site is marked in Blue
His tag is marked in GREEN

Supplementary Figure 12

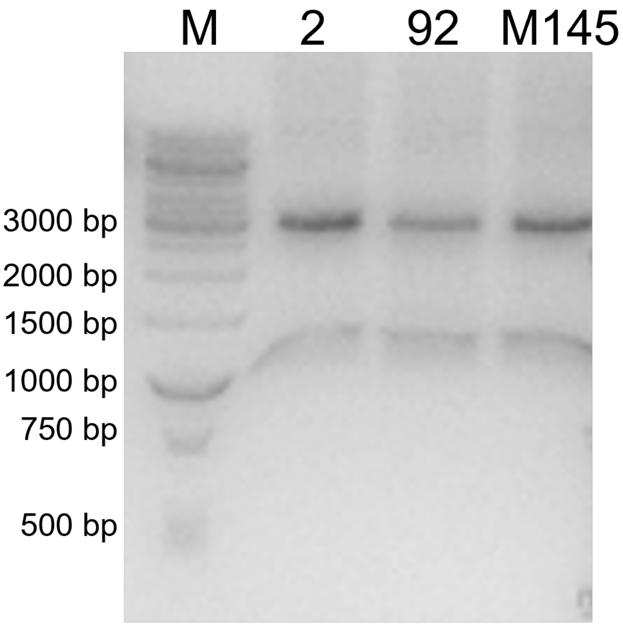

Figure 12: ***Digestion analysis of lipA genes in pSET152***
Restriction analysis with NdeI and BamHI expected size gene 1250 bp.

Supplementary Table 7

Table 7: ***Obtained Streptomyces strains***

Organism	Construct	Function	Working name
S. coelicolor M145	-	WT	WT
S. coelicolor M145 Δ*lipA*	-	Knock-out	KO
S. coelicolor M145 Δ*lipA*	pSET152_pGAP_*lipA* M145	Overexpression	S3
S. coelicolor M145 Δ*lipA*	pSET152_pGAP_*lipA* MBT2	Overexpression	S5
S. coelicolor M145 Δ*lipA*	pSET152_pGAP_*lipA* MBT92	Overexpression	S7

Supplementary Table 8

*Table 8: **Primers for amplification and cloning of PET46***

name	sequence
JA_F_PET46	atatacatatgattaagccggtgacctt
JA_R_PET46_stop BamHI	GTATACGGATCCttactcgagtgcggccg

Supplementary Note 3

Codon DNA sequences for protein expression in *E. coli BL21*

PETase
ATGCAGACCAATCCGTATGCACGTGGTCCGAATCCGACCGCAGCAAGCCTGGAAGCAA
GCGCAGGTCCGTTTACCGTTCGTAGCTTTACCGTTAGCCGTCCGAGCGGTTATGGTGCA
GGCACCGTTTATTATCCGACCAATGCCGGTGGCACCGTTGGTGCAATTGCCATTGTTCC
GGGTTATACCGCACGTCAGAGCAGCATTAAATGGTGGGGTCCGCGTCTGGCAAGCCATG
GTTTTGTTGTTATTACCATTGATACCAACAGCACCCTGGATCAGCCGAGCAGCCGTAGCA
GTCAGCAGATGGCAGCACTGCGTCAGGTTGCCAGCCTGAATGGCACCAGCAGCAGCCC
GATTTATGGTAAAGTTGATACAGCACGTATGGGTGTTATGGGTTGGAGCATGGGTGGTGG
TGGTAGCCTGATTAGCGCAGCAAATAATCCGAGCCTGAAAGCAGCAGCACCGCAGGCTC
CGTGGGATAGCAGCACCAATTTTAGCAGCGTTACCGTTCCGACACTGATTTTTGCATGTG
AAAATGATAGCATTGCACCGGTTAATAGCAGCGCACTGCCGATCTATGATAGTATGAGCC
GTAATGCAAAACAGTTTCTGGAAATTAATGGTGGCAGCCATAGCTGTGCAAATAGCGGTA
ATAGCAATCAGGCACTGATCGGTAAAAAGGGTGTTGCATGGATGAAACGCTTTATGGATA
ATGATACCCGCTATAGCACCTTTGCCTGCGAAAATCCGAATAGCACCCGTGTTAGCGATT
TTCGTACCGCAAATTGTAGCTAA

PET40
ATGGCCGAAAATCCGTATGAACGTGGTCCGGCACCGACCACCAGTAGCATTGAAGCAAG
CCGTGGTAGCTTTGCAACCAGCACCGTTACCGTTAGCCGTCTGGCAGTTAGCGGTTTTG
GTGGTGGCACCATCTATTATCCGACCAGCACCACCGCAGGCACCTTTGGTGCAATTAGC
ATTGCACCGGGTTTTACCGCACTGCAGAGCAGCATTGCATGGCTGGGTCCGCGTCTGG
CAAGCCAGGGTTTTGTTGTTTTTACCATTGATACCCTGACCACCTCAGATCAGCCGGATA
GCCGTGGTCGTCAGCTGCTGGCAGCACTGGATTATCTGACCCAGCAGAGCAGTGTTCG
TAGCCGTATTGATAGCAGTCGTCTGGGTGTTGTTGGTCATAGCATGGGTGGTGGTGGTAC
ACTGGAAGCAGCACGTAGCCGTCCGAGTCTGCAGGCAGCAATTCCGCTGACCGGTTGG
AATCTGACCAAAACCTGGTCAACCGTTCGTGTTCCGACACTGGTTGTTGGTGCACAGGC
AGATACCGTTGCACCGGTTGCAAGCCATAGCATTCCGTTTTATAACAGCCTGCCGAGCAG
CCTGGATAAAGCATATCTGGAACTGCGTGGTGCCAGCCATTTTGCACCGAATAGCAGCAA
TACCACCATTGCAAAATATACCCTGAGCTGGCTGAAACGCTTTATTGATAATGATACCCGC
TATGAGCAGTTTCTGTGTCCGATTCCGAGCACCAGCCTGAGCATTAGCGATTATCGTGGT
AATTGTCCGCATAACGGTTAA

PET46
ATGATTAAGCCGGTGACCTTTATGTCAGAAGGTGAACAGATTATTGGCGTGCTGCATGTT
CCGGATGATCTGCGCGGCGATAAACGCGCCCCGGCCATTGCCATGTTTCATGGCTTTAC
CGGCAATAAATCAGAAGCACATCGTCTGTTTGTTCATGTTGCACGCTCACTGTGTAATGAT
GGCTTTGTGGTGCTGCGCTTTGATTTTCGTGGCAGCGGCGATAGTGATGGCGAATTTGA
AGATATGACCGTTCCGGGCGAAGTGTGTGATGCCTCTCGCTCTATTGATTTTCTGTCAGA
ACTGAATTTTGTGGATAGCGAACGCATTGGCGTGCTGGGCCTGAGTATGGGTGGTCGCG
TTGCAGCCATTTTAGCAAGCAAAGATCGTCGCATTAAATTTGTTATTCTGTATAGTGCAGC
CTTAACCCCGCTGCGCCGCAAATTTCTGGAAGGCTTAGAAAAAGAATCAATTCGTCGCCT
GGAAATGGGCGAAGCAGTTCATGTGGGTAATGGTTGGTATCTGAAAAAAGGCTTTTTTGA
AACCGTGGATAGTATTGTGCCGTTAGATGTGCTGGATCGCATTCGCGTTCCGGTTTTAATT
ATTCATGGCGATAGCGATTCAGTTATTCCGTTAGATGGTGCACTGAAAGGCTATGAAATTA
TTCGTGATCTGAATGATAAAAATGAACTGTATATTGTTCGCGGCGGTGATCATGTGTTTAC
CCGCCGCGAACATACCATTGAAGTTATTGAACGTACCCTGGATTGGTTACGTAGCTTAAAT
CTGGTCGACAAGCTTGCGGCCGCACTCGAG

LCC
ATGCAGAGCAATCCGTATCAGCGTGGTCCGAATCCGACACGTAGCGCACTGACCGCAGATGGTCCGTTTAGCGTT
GCAACCTATACCGTTAGCCGTCTGAGCGTTAGCGGTTTTGGTGGTGGTGTTATCTATTATCCGACCGGCACCAGCC
TGACCTTTGGTGGTATTGCAATGAGTCCGGGTTATACAGCAGATGCAAGCAGCCTGGCATGGCTGGGTCGTCGTC
TGGCAAGCCATGGTTTTGTTGTTCTGGTGATTAATACCAACAGCCGCTTTGATTATCCGGATTCACGTGCAAGCCA
GCTGAGCGCAGCACTGAATTATCTGCGTACCAGCAGTCCGAGCGCAGTTCGTGCACGTCTGGATGCAAATCGTCT
GGCCGTTGCAGGTCATAGCATGGGTGGCGGTGGCACCCTGCGTATTGCAGAACAGAATCCGAGCCTGAAAGCA
GCAGTTCCGCTGACACCGTGGCATACCGATAAAACCTTTAATACCAGCGTTCCGGTTCTGATTGTTGGTGCAGAA
GCAGATACCGTTGCACCGGTTAGCCAGCATGCAATTCCGTTTTATCAGAATCTGCCGAGCACCACACCGAAAGTT
TATGTTGAACTGGATAATGCCAGCCATTTTGCACCGAATAGCAATAATGCAGCGATTAGCGTTTATACCATCAGCTG
GATGAAACTGTGGGTTGATAATGATACCCGTTATCGTCAGTTTCTGTGCAATGTTAATGATCCGGCACTGAGCGAT
TTTCGTACCAATAATCGTCATTGTCAGTAA

TfCut2
GCCAATCCGTATGAACGTGGTCCGAATCCGACCGATGCACTGCTGGAAGCACGTAGCGGTCCGTTTAGCGTTAGC
GAAGAAAATGTTAGCCGTCTGAGCGCAAGCGGTTTTGGTGGTGGCACCATCTATTATCCGCGTGAAAATAACACC
TATGGTGCAGTTGCAATTAGTCCGGGTTATACCGGCACCGAAGCAAGCATTGCATGGCTGGGTGAACGTATTGCA
AGCCATGGTTTTGTTGTGATTACCATTGATACCATTACCACACTGGATCAGCCGGATAGCCGTGCAGAACAGCTGA
ATGCAGCACTGAATCACATGATTAATCGTGCAAGCAGCACCGTTCGTAGCCGTATTGATAGCAGCCGTCTGGCAG
TTATGGGTCATAGCATGGGTGGTGGTGGTAGCCTGCGTCTGGCAAGCCAGCGTCCGGATCTGAAAGCAGCAATT
CCGCTGACACCGTGGCATCTGAACAAAAATTGGAGCAGCGTTACCGTTCCGACACTGATTATTGGTGCAGATCTG
GATACCATTGCACCGGTTGCGACCCATGCAAAACCGTTTTATAACAGCCTGCCGAGCAGCATTAGCAAAGCATATC
TGGAACTGGATGGTGCAACCCATTTTGCACCGAATATTCCGAATAAAATCATCGGCAAATATAGCGTTGCCTGGCT
GAAACGTTTTGTGGATAATGATACCCGTTATACCCAGTTTCTGTGTCCGGGTCCGCGTGATGGTCTGTTTGGTGAA
GTTGAAGAATATCGTAGCACCTGTCCGTTT

2LipA
ATGGCCGATAATCCGTATGAACGTGGTCCGGCACCGACCGAAAGCAGCATTGAAGCACTGCGTGGTCCGTATAGC
GTTGCAGATACCAGCGTTAGCAGCCTGGCAGTTACCGGTTTTGGTGGTGGCACCATCTATTATCCGACCAGCACC
AGTGATGGCACCTTTGGTGCAGTTGTTATTAGTCCGGGTTTTACCGCATATCAGAGCAGTATTGCATGGCTGGGTC
CGCGTCTGGCAAGCCAGGGTTTTGTTGTTTTTACCATTGATACCAATACCACACTGGATCAGCCGGATAGCCGTG
GTCGTCAGCTGCTGGCAGCACTGGATTATCTGACCGAACGTAGCAGCGTTCGTGGTCGTATTGATAGCAGCCGTC
TGGGTGTTATGGGTCATAGCATGGGTGGTGGTGGTAGCCTGGAAGCAGCAAAAAGCCGTCCGAGTCTGCAGGC
AGCAATTCCGCTGACACCGTGGAATCTGGATAAAAGCTGGCCTGAAGTTAGCACCCCGACACTGATTGTTGGTGC
AGATGGTGATACCGTTGCACCGGTGAGCAGCCATAGCGAACCGTTTTATAGCAGTCTGCCGAGCGGCACCGATC
GTGCATATCTGGAACTGAATAATGCAACCCATTTTAGCCCGAATACCAGCAATACCACCATTGCCAAATATAGCATT
AGCTGGCTGAAACGCTTCATCGATAATGATACCCGTTATGAACAGTTTCTGTGTCCGCTGCCTCGTCCGAGCCTGA
CCATTGAAGAATATCGTGGTAATTGTCCGCATGGCAGCTAA

92LipA
ATGGCCGATAATCCGTATGAACGTGGTCCGGCACCGACCGAAAGCAGCATTGAAGCACTGCGTGGTCCGTATAGC
GTTGCAGATACCCGTGTTAGCAGCCTGGCAGTTACCGGTTTTGGTGGTGGCACCATCTATTATCCGACCAGCACC
AGTGATGGCACCTTTGGTGCAGTTGCCATTGCACCGGGTTTTACCGCATATCAGAGCAGCATGGCATGGCTGGGT
CCGCGTCTGGCAAGCCAGGGTTTTGTTGTTTTTACCATTGATACCAATACCACACTGGATCAGCCGGATAGCCGTG
GTCGTCAGCTGCTGGCAGCACTGGATTATCTGACCGAACGTAGCAGCGTTCGTGGTCGTATTGATAGCAGCCGTC
TGGGTGTTATGGGTCATAGCATGGGTGGTGGTGGTACACTGGAAGCAGCAAAAAGCCGTCCGAGTCTGCAGGC
AGCAATTCCGCTGACACCGTGGAATCTGGATAAAAGCTGGCCTGAAGTTAGCACCCCGACACTGATTGTTGGTGC
AGATGGTGATACCATTGCTCCGGTTGCAAGCCATGCAGAACCGTTTTATAGCGGTCTGCCGAGCGCAACCGATCG
TGCATATCTGGAACTGAATGGTGCAACCCATTTTAGCCCGAATAGCAGCAATACCACCATTGCAAAATATAGCGTT
AGCTGGCTGAAACGCTTCATTGATAATGATACCCGCTATGAACAGTTTCTGTGTCCGCTGCCTCGTCCGAGCCTGA
CCATTGAAGAATATCGTGGTAATTGTCCGCATGGCAGCTAA

*Sc*LipA
ATGGCCGATAATCCGTATGAACGTGGTCCGGCACCGACCGAAAGCAGCATTGAAGCACTGCGTGGTCCGTATAGC
GTTGCAGATACCAGCGTTAGCAGCCTGGCAGTTACCGGTTTTGGTGGTGGCACCATCTATTATCCGACCAGCACC
AGTGATGGCACCTTTGGTGCAGTTGTTATTGCACCGGGTTTTACCGCATATCAGAGCAGTATTGCATGGCTGGGT
CCGCGTCTGGCAAGCCAGGGTTTTGTTGTTTTTACCATTGATACCAATACCACACTGGATCAGCCGGATAGCCGTG
GTCGTCAGCTGCTGGCAGCACTGGATTATCTGACCGGTCGTAGCAGCGTTCGTGGTCGTATTGATAGCGGTCGTC
TGGGTGTTATGGGTCATAGCATGGGTGGTGGTGGTACACTGGAAGCAGCAAAAAGCCGTCCGAGTCTGCAGGC
AGCAATTCCGCTGACACCGTGGAATCTGGATAAAAGCTGGCCTGAAGTTAGCACCCCGACACTGGTTGTTGGTG
CAGATGGTGATACCATTGCTCCGGTTGCAAGCCATGCAGAACCGTTTTATAGCGGTCTGCCGAGCAGCACCGATC
GTGCATATCTGGAACTGAATAATGCAACCCATTTTAGCCCGAATACCAGCAATACCACCATTGCCAAATATAGCATT
AGCTGGCTGAAACGCTTTATCGATGATGATACCCGTTATGAACAGTTTCTGTGTCCGCTGCCTCGTCCGAGCCTGA
CCATTGAAGAATATCGTGGTAATTGTCCGCATGGCAGCTAA

Supplementary Table 9

*Table 9: **Plasmids for expression Lip A variants in E. coli***

Plasmids	Characteristics	source
pMA_LipA_MBT2	Amp^R, LipA_MBT2	Thermofisher
pMA_LipA_MBT92	Amp^R, LipA_MBT92	Thermofisher
pMA_LipA_M145	Amp^R, LipA_M145	Thermofisher
pET16b_ LipA_MBT2	Amp^R,His-tag, LipA_MBT2	This work
pET16b_LipA_MBT92	Amp^R,His-tag, LipA_MBT92	This work
pET16b_ LipA_M145	Amp^r,His-tag, LipA_M145	This work
pET16b_LCC	Amp^R,His-tag, LCC	This work
pET16b_PET40	Amp^R,His-tag, PET40	This work
pET16b_PET46	Amp^r,His-tag, PET46	This work
pET16b_PETase	Amp^R,His-tag, PETase	This work
pET16b_TfCut2	Amp^r,His-tag, TfCut2	This work

Supplementary Table 10

Table 10: ***E. coli* strains used in this study.**

Strain	Plasmid	Purpose
E. coli BL21	pET16b	Negative control
	pET16b_LCC	Positive control Cutinase activity
	pET16b_PET40	Closest homologue
	pET16b_PET46	Positive control BHET/ MHET activity
	pET16b_PETase	Positive control PETase activity
	pET16b_TfCut2	Positive control Cutinase activity
	pET16b _LipA M145	
	pET16b _LipA MBT2	
	pET16b _LipA MBT92	

Supplementary Figure 13

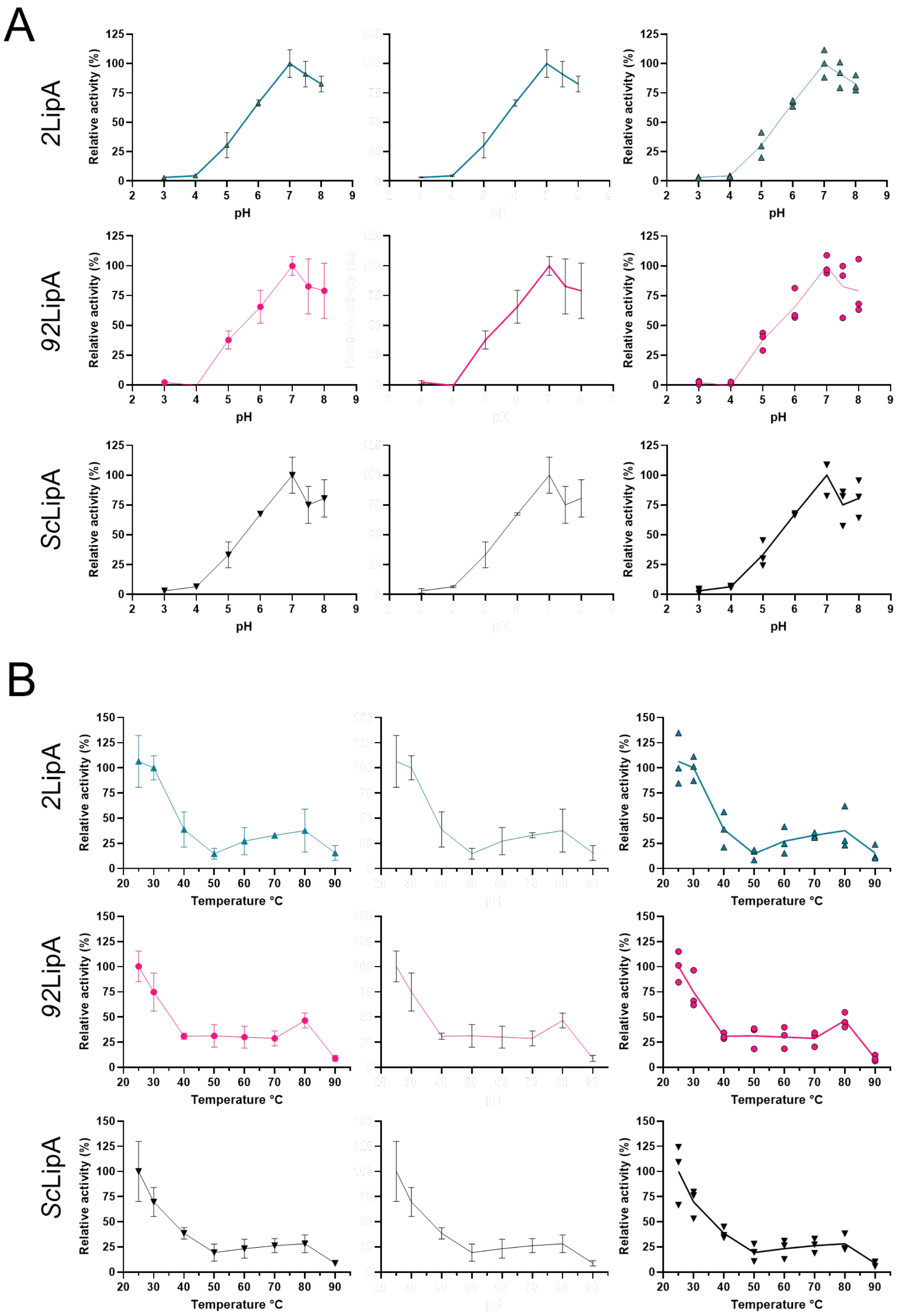

Figure 13: ***Original Graph used for the Determination of optimal enzyme conditions***
A) relative activity on para-nitrophenol dodecanoate at different pH for S2LipA, S92LipA and ScLipA B) relative activity on para-nitrophenol dodecanoate at different temperatures for S2LipA , S92LipA and ScLipA. For all graphs n=3, from left to right the graphs display the error bars (SD) and mean, the middle graphs display the error

bars only and the right graphs display the individual measurements. The, graphs in figure 7 are an overlay of the middle and right graph.
