# Supplementary material for: Polyester degradation by soil bacteria: identification of conserved BHETase enzymes in Streptomyces
Source: Commun Biol. 2024 Jun 12;7:725. doi: 10.1038/s42003-024-06414-z (PMC11169514; doi:10.1038/s42003-024-06414-z)
Supplement: Supplementary file 3 — Description of Additional Supplementary Files [file 42003_2024_6414_MOESM3_ESM.docx]

**Description of Additional Supplementary Files**

**File name:** Supplementary Data 1

**Description:** The source data and statistics of figure 2

**File name:** Supplementary Data 2

**Description:** The source data and statistics of figure 4

**File name:** Supplementary Data 3

**Description:** The source data and statistics of figure 7

**File name:** Supplementary Movie 1

**Description:** Time lapse video of the negative control of BHET without strain

**File name:** Supplementary Movie 2

**Description:** Time lapse video of BHET with wildtype S. coelicolor M145

**File name:** Supplementary Movie 3

**Description:** Time lapse video of BHET with S. coelicolor M145 ΔlipA

**File name:** Supplementary Movie 4

**Description:** Time lapse video of BHET with S. coelicolor S3

**File name:** Supplementary Movie 5

**Description:** Time lapse video of BHET with S. coelicolor S5

**File name:** Supplementary Movie 6

**Description:** Time lapse video of BHET with S. coelicolor S7
